# Beyond Bacterial Causes: A Case of Recurrent Urinary and Vulvovaginal Symptoms Due to *Enterobius vermicularis*—Case Report

**DOI:** 10.1155/crdi/9586077

**Published:** 2025-10-29

**Authors:** Momin Khan, Safeena Khan, Muhammad Haris Khan, Umar Tariq, Wajeeh Ur Rehman

**Affiliations:** ^1^Department of Medicine, Saidu Teaching Hospital, Swat, Pakistan; ^2^Department of Medicine, Khyber Medical College, Peshawar, Pakistan; ^3^Department of Medicine, Saidu Medical College, Swat, Pakistan

**Keywords:** *Enterobius vermicularis*, pediatric, urinary tract infection, vulvovaginitis

## Abstract

Bacterial and fungal infections are recognized as prevalent etiological factors contributing to the symptoms associated with urinary tract infections and vulvovaginitis, respectively. This report presents a notable instance of a nonbacterial cause, specifically involving *Enterobius vermicularis* (pinworm). A 7-year-old female patient from a low socioeconomic background is reported, presenting with a 14-month history of recurrent urinary symptoms and vulvovaginitis. This clinical picture persists despite multiple courses of antibiotic therapy and negative urinary cultures. The diagnosis was established following meticulous urine collection, which demonstrated the presence of motile organisms identified as *Enterobius vermicularis*. The patient exhibited a favorable response to the administered treatment. This case highlights the importance of considering parasitic infections in the differential diagnosis of recurrent urinary symptoms, especially in pediatric patients with inadequate hygiene and lower socioeconomic conditions.

## 1. Introduction

*Enterobius vermicularis*, commonly referred to as pinworms, represents the most common human parasite. It is the leading helminth infection documented in the United States and is exclusively transmissible among humans [1]. The condition exhibits a global prevalence, with crowded environments identified as a significant risk factor for infection. Furthermore, it is observed to be somewhat more prevalent in developing nations [2, 3].

Pinworm infection is initiated by accidental ingestion of infective eggs, which hatch in the small intestine and develop into adult worms in the caecum [4]. The gastrointestinal system is the most common location of invasion [5].

Traditionally acknowledged for its association with enterobiasis, emerging research has underscored its possible involvement in extraintestinal manifestations, notably urinary tract infections (UTIs) [6]. UTIs are common pediatric diseases rarely attributed to nonbacterial causes, such as parasite infections, which can manifest with symptoms that complicate diagnosis and treatment, frequently resulting in complications if not addressed appropriately [7]. This case is particularly noteworthy due to the limited documentation in the literature regarding the association between *Enterobius vermicularis* and UTIs or other vulvovaginal symptoms. Heightened understanding of *Enterobius vermicularis* and its unusual manifestations might enhance prompt and effective care strategies, hence improving patient outcomes.

## 2. Case Presentation

A 7-year-old girl from a low socioeconomic family exhibited a 14-month history of urinary tract symptoms, comprising dysuria, urinary urgency, and intermittent hematuria, without fever. She was experiencing severe vulvar pruritus and discomfort. She additionally reported the intermittent excretion of small, worm-like structures in her urine. Despite consultations with numerous healthcare professionals and treatment with several antibiotics, including ciprofloxacin, cefixime, ceftriaxone, and cefoperazone sulbactam, her symptoms continued unabated. Two sets of urine cultures were previously performed, yielding negative results with no bacterial growth. A female physician conducted a genital examination, which revealed a red, irritated vulva without any discharge. A Scotch tape test conducted at that time did not disclose any indications of parasite infection.

**Figure 1:**
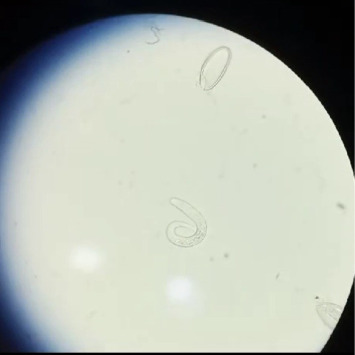
Microscopic image showing an adult worm and its ovum.

To aid in diagnosis, the patient was directed to collect urine in a transparent container to obtain the worm-like creatures for further analysis, ensuring that only the surface urine was removed if required. A follow-up visit was scheduled on the third day, which included a repeat Scotch tape test and urine sample collection. Upon her visit on the third day, she presented with identical clinical characteristics. In the final stage of urine sample collection, identification of *Enterobius vermicularis* was performed through microscopic examination of urine sediment. This process commonly involves centrifuging the urine specimen, followed by analysis of the sediment under a light microscope to detect eggs, larvae, or adult worms. A thorough analysis of the urine sample indicated the presence of 8-9 motile organisms, which were subsequently identified by microbiological testing as *Enterobius vermicularis*, leading to a diagnosis of pinworm infection. The adult worm and its ovum are visualized in Figure 1.

The patient was then treated with a 3-day course of mebendazole as part of her treatment regimen. At the 2-week follow-up, she presented without any symptoms, exhibiting normal examination findings and a negative result on the Scotch tape test. Furthermore, a single Scotch tape test was performed on her siblings as well. The results indicated that one sibling tested positive and subsequently received the same treatment regimen, thereby ensuring comprehensive management of the infection within the family.

## 3. Discussion

This case illustrates how extraintestinal *Enterobius vermicularis* infection can be mistaken for bacterial UTI, resulting in 14 months of misdiagnosis and unnecessary antibiotic exposure. Repeated ineffective antibiotic regimens and persistently negative urine cultures underscore the imperative to include parasitic infections in the differential diagnosis of pediatric urogenital symptoms, especially in populations from lower socioeconomic status in which enterobiasis is more frequent [8, 9].

Urogenital involvement is characteristically more frequent among females, an observation attributable to the minimal distance separating the anal and urogenital orifices [6, 10, 11]. The present case aligns with previously documented reports that list dysuria, urgency, and hematuria as salient urogenital symptoms [6, 10]. Additional studies have corroborated the emergence of extended clinical courses, consistently aseptic urine cultures, and the lag in securing an accurate diagnosis of parasitic origin [6, 10].

This case is distinguished by the patient's age of 7 years and by the direct observation of motile *Enterobius vermicularis* within freshly voided urine specimens [6, 12]. Typical presentations show eggs or nonviable parasites predominantly within the extraurethral tissues; hence, the direct viewing of motile *Enterobius vermicularis* is a rare and clinically significant finding [6, 10, 11]. The prevailing body of evidence indicates that ascending migration of the pathogen via the urethra constitutes the most probable pathogenetic mechanism, a conclusion further corroborated by the present case, which exhibits clear evidence of direct involvement of the urethra rather than an infection disseminated through the bloodstream [11, 12].

The standard negative Scotch tape test underscores the inherent constraints of the conventional method, achieving a sensitivity of only 50% across detected infections [12, 13]. In contrast, polymerase chain reaction (PCR)–driven diagnostics markedly enhance sensitivity; nested PCR identifies as much as 88.9% of clinical cases, a substantial improvement over the Scotch tape test [13, 14]. Moreover, real-time PCR technology permits the identification of DNA from single ova, concurrently offering high specificity [13, 14].

PCR approaches, despite their analytical precision, necessitate laboratory-grade apparatus, skilled technologists, and uninterrupted electrical supply, prerequisites frequently unmet in primary-care sites serving low socioeconomic populations. In settings where resources are limited, feasible alternatives comprise conducting Scotch tape tests over three consecutive mornings (yielding 90% sensitivity), collecting early-morning specimens, and equipping primary-care providers with training focused on the recognition of clinical presentations [14]. Alternatively, the direct microscopic examination of fresh urine specimens, as executed in this study, offers on-the-spot confirmation whenever motile parasites are visible [6, 10].

An extended clinical course entails substantial patient distress coupled with avoidable expenditure of healthcare resources [8, 12]. Systematic family screening was decisive, identifying one asymptomatic sibling, thereby underscoring the urgent need for household-centered management strategies in light of documented high intrafamilial transmission [8, 9, 12].

Successful mebendazole treatment that led to complete resolution of symptoms is consistent with the expected therapeutic efficacy of the drug against enteric helminths [8, 12]. From a public health standpoint, the present case exemplifies the systemic obstacles encountered in the management of parasitic diseases among populations experiencing restricted access to confirmatory diagnostic tools. Care providers must refine their clinical reasoning to distinguish among infectious causes reliably, thereby preventing unwarranted antibiotic prescriptions. This case illustrates how heightened diagnostic accuracy yields immediate benefit to the individual patient and advances the broader objective of antimicrobial stewardship.

Healthcare professionals must receive rigorous training on differentiating parasitic infections from bacterial diseases to avert the overprescription of antibiotics and to reduce the lag in producing definitive diagnoses. This diagnostic challenge shows us an educational gap where parasitic diseases are not getting enough attention next to bacterial infections, especially in areas where both are common. Expanded training programs must prioritize thorough documentation of patients' relevant social histories, systematic identification of atypical symptoms, and the application of validated diagnostic algorithms directed toward cases where conventional bacterial cultures fail to yield positive findings.

## Data Availability

The data supporting the findings of this case report are available from the corresponding author upon reasonable request.
